# Revealing the factors influencing a fermentative biohydrogen production process using industrial wastewater as fermentation substrate

**DOI:** 10.1186/s13068-014-0139-1

**Published:** 2014-09-24

**Authors:** Iulian Zoltan Boboescu, Mariana Ilie, Vasile Daniel Gherman, Ion Mirel, Bernadett Pap, Adina Negrea, Éva Kondorosi, Tibor Bíró, Gergely Maróti

**Affiliations:** Polytechnic University of Timisoara, Timisoara, Romania; Seqomics Biotechnology Ltd, Szeged, Hungary; Hungarian Academy of Sciences, Biological Research Centre, Temesvari krt. 62., Szeged, 6726 Hungary; Szent István University, Faculty of Economics, Agricultural and Health Studies, Szarvas, Hungary

**Keywords:** Biohydrogen, Central composite experimental design, Microbial inocula, Metagenomics, Industrial wastewater

## Abstract

**Background:**

Biohydrogen production through dark fermentation using organic waste as a substrate has gained increasing attention in recent years, mostly because of the economic advantages of coupling renewable, clean energy production with biological waste treatment. An ideal approach is the use of selected microbial inocula that are able to degrade complex organic substrates with simultaneous biohydrogen generation. Unfortunately, even with a specifically designed starting inoculum, there is still a number of parameters, mostly with regard to the fermentation conditions, that need to be improved in order to achieve a viable, large-scale, and technologically feasible solution. In this study, statistics-based factorial experimental design methods were applied to investigate the impact of various biological, physical, and chemical parameters, as well as the interactions between them on the biohydrogen production rates.

**Results:**

By developing and applying a central composite experimental design strategy, the effects of the independent variables on biohydrogen production were determined. The initial pH value was shown to have the largest effect on the biohydrogen production process. High-throughput sequencing-based metagenomic assessments of microbial communities revealed a clear shift towards a *Clostridium* sp*.*-dominated environment, as the responses of the variables investigated were maximized towards the highest H_2_-producing potential. Mass spectrometry analysis suggested that the microbial consortium largely followed hydrogen-generating metabolic pathways, with the simultaneous degradation of complex organic compounds, and thus also performed a biological treatment of the beer brewing industry wastewater used as a fermentation substrate.

**Conclusions:**

Therefore, we have developed a complex optimization strategy for batch-mode biohydrogen production using a defined microbial consortium as the starting inoculum and beer brewery wastewater as the fermentation substrate. These results have the potential to bring us closer to an optimized, industrial-scale system which will serve the dual purpose of wastewater pre-treatment and concomitant biohydrogen production.

## Background

Our society today increasingly requires more energy to maintain overall ascending economic trends. Although the demand for energy is permanently growing, the reserves of our primary energy carriers will be depleted within a few decades [1]. In addition, our fossil fuel-based economy is dramatically accelerating the process of global warming with severe and permanent consequences for the environment [2]. Therefore, novel and safe energy carriers must be introduced. Hydrogen satisfies all the requirements for a clean and renewable fuel, producing only water as a by-product upon combustion or direct use in fuel-cell technology. Hydrogen has the highest energy content per unit weight of any known fuel (142 kJ/g or 61,000 Btu/lb) and can be transported for domestic/industrial consumption through conventional means [3,4]. In addition to this, H_2_ gas is safer to handle than domestic natural gas, and can be used directly in internal combustion engines or in fuel cells to generate electricity [3]. Hydrogen use in fuel cells is inherently more efficient than the combustion currently required for the conversion of other potential fuels to mechanical energy [5]. However, most hydrogen is currently produced by conventional chemical or electrolytic methods, which require high amounts of energy and expensive technologies [6].

In the last few years, attention has shifted towards novel and less energy-intensive technologies for producing hydrogen [7-9]. Among the various hydrogen production processes, the biological methods (direct and indirect photolysis, photo-fermentation, and dark fermentation) appear to be the most promising [10-12]. In addition, certain methods of producing biological hydrogen such as dark fermentation can utilize various organic wastes as a substrate for fermentative hydrogen production, thus coupling organic waste treatment with renewable energy generation [13-16]. Recently, reducing the cost of wastewater treatment and finding ways to produce useful products from wastewater has been gaining importance with regard to environmental sustainability. One way to address both issues is to simultaneously generate bioenergy in the form of hydrogen by utilizing the organic matter present in wastewater [17]. In addition, certain types of wastewater generated by various industrial processes are considered ideal substrates because they contain high levels of easily degradable organic material [18].

The complete oxidation of glucose could yield a theoretical maximum of 12 moles of H_2_ per mole of glucose, but in this case no energy can be utilized to support the growth and metabolism of the hydrogen-producing organism [7,19,20]. Strictly anaerobic hydrogen-producing microbes are able to generate a maximum of 4 moles of H_2_ per mole of degraded glucose [21-23]. Fermentative H_2_ production using complex microbial communities therefore has the advantage of a high hydrogen production rate utilizing complex organic wastes as fermentation substrates with limited amounts of additional external energy input [24]. Bacteria and other microbes capable of hydrogen production exist widely in natural environments rich in organic nutrients such as soil, wastewater sludge, and compost [25-27]. Microbial populations sampled from these habitats can thus be used as cheap and highly efficient inocula for fermentative hydrogen production. In addition, dark hydrogen production processes using mixed microbial cultures as starting inocula are more efficient than those using pure cultures. The reason is that mixed cultures represent more simple systems to operate which are easier to control, and may be able to degrade a broader range of feedstock [28]. The use of mixed microbial cultures as starting inocula also allows the use of unsterile fermentation substrates, such as most types of wastewater. However, in a fermentative hydrogen production process using mixed cultures, the hydrogen produced by hydrogen-evolving bacteria can be consumed by hydrogen-consuming bacteria [29,30]. Strategies for pretreatment of the parent inocula are therefore required to restrict or even terminate the methanogenic process to ensure that H_2_ remains the end product in the metabolic flow [31-33].

One of the major impediments in developing biohydrogen (bioH_2_) production processes for commercialization is low hydrogen production yield. The biological production rate of hydrogen and its molar yield, like most other bioprocesses, are dependent on several parameters including the activity rate of hydrogen-producing and -consuming bacteria, substrates, inorganic nutrients, and operational conditions of the bioreactor, among other factors [34]. Identifying these influencing factors and optimizing the fermentation conditions, particularly the nutritional and environmental parameters, is thus of primary importance in the bioprocess development. The most widely used screening and optimization strategy is the design of experiment (DOE) method, by which certain factors are selected and deliberately varied in a controlled manner, in order to study their effects, facilitate process comprehension, and even to improve performance [35-43]. The use of such DOE methods for process optimization in fermentative hydrogen production processes is critical due to the dynamic and complex nature of these systems.

In the present study, a central composite experimental design was used to investigate the influence of the process variables involved in batch-mode biohydrogen production, as well as to optimize their response. The experiments were performed using a defined mixed microbial consortium as the starting inoculum and wastewater obtained from a beer-brewing factory as the fermentation substrate. High-throughput metagenomic microbial community assessments as well as analysis of substrate degradation rates were performed during the experiments to understand the fermentation mechanisms involved. The results of this study have the potential to propel us closer to achieving and optimizing an industrial-scale system which will serve the dual purpose of providing wastewater pretreatment coupled with biohydrogen production.

## Results and discussion

Batch experiments were conducted using mixed microbial consortia able to degrade complex organic substrates like wastewater in addition to simultaneous biohydrogen production. The involvement of biological, chemical, and physical factors influencing the fermentative biohydrogen production process as well as the interactions of these parameters were assessed by experiments designed by statistics-based methods and metagenomic monitoring of this complex ecosystem.

### Effect of various influencing factors and the interactions between them on the biohydrogen production process

**Figure 1:**
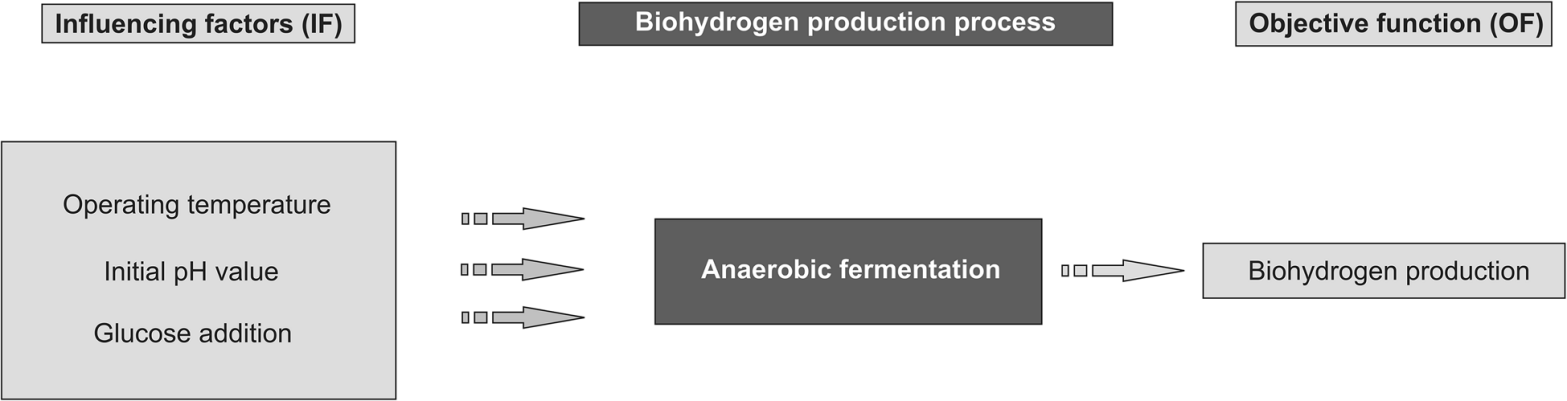
**Cybernetic representation of the investigated dark biohydrogen fermentation process with an input-output structure.** The anaerobic fermentation of the wastewater is considered a black-box system influenced by different variables, with consequences on the objective function (in this case, bioH_2_ production).

To assess the influence of various factors on the biohydrogen production process, as well as the level of interaction between these factors, a cybernetic representation of the dark biohydrogen fermentation process was developed, with an input-output structure (Figure 1). The input data are defined as influencing factors (IF) while the system output data are defined as objective functions (OF). By developing and applying this experimental modeling approach, connecting relationships (most frequently with a predefined polynomial shape) between the IF and the OF (in this case, H_2_ production rates) can be identified for an experimental domain previously defined as being of interest. The data obtained by applying this experimental design strategy can be used to generate predictions regarding the behavior of the system under investigation. They can also provide deep insight into the degree of influence of the investigated variables on the objective functions. In addition, by applying advanced statistical approaches, the optimum region of a system for a specific response can be identified (in this case, biohydrogen production rate).

Because of the steep curve generated by the response surface approach during the investigation of an optimal area within a specific system, using linear methods to adequately identify the stationary point becomes obsolete. Therefore, depending on the number of investigated variables, further additional experimental points are required. One of the most recommended approaches to address this issue is the central composite experimental design method. By applying this strategy, one can use the information obtained through a first-order mathematical model, completing these insights by consecutively extending the experimental approach to explain a second-order mathematical model. Thus, a central composite design (CCD) consists of a standard first-order design with a *2*^*k*^*or 2*^*k-p*^ orthogonal factorial point, where k is the number of selected factors and p the number of interactions replaced with influencing factors. *2*^*k*^ axial points are displayed in a "star pattern" at distance α from the center of the design with *n*_0_ center points. Based on preliminary screening experiments, three IF were selected; fermentation temperature, starting pH value, and degradable substrate availability (glucose addition; Table 1). Each IF was defined between two physical values, low and high, coded as -1 and +1 respectively, with a center value coded as 0.

**Table 1:** **Physical and coded values of the variables used in the central composite design**

**Coded symbol**	**Variables**	**Values of coded levels**				
		**(-1.28)**	**(-1)**	**(0)**	**(+1)**	**(+1.28)**
X_1_	Operating temperature (°C)	23.28	25	31	37	38.72
*X*_2_	Initial value of fermentation pH	4.56	4.8	5.65	6.5	6.74
X_3_	Glucose addition (g/L)	3.56	5	10	15	16.44

The relationship between the physical and coded values is given by the following equation:

1 $$ {x}_{jcod}=\frac{x_{jphys}-{x}_{j0 phys}}{I_{jphys}},\ j=1,2,3 $$

where:

- x_jcod_ is the coded value of the j factor
- x_jphys_ is the physical value of the j factor
- x_j0phys_ is the central level of the j factor
- I_jphys_ is the variation interval for the j factor; double this value gives the variation range D (Equation 2)

2 $$ D={x}_{jphys\  high}-{x}_{jphys\  low}=2\ {I}_{jphys} $$

The factorial portion of the CCD is a complete 2^3^ factorial with eight runs, which contains all the possible combinations within the defined levels of the investigated variables (runs 1-8; Table 2). The additional experimental runs (9-14) represent supplementary axial points displayed in a "star pattern" around the center of the design, at distance α of 1.287 from the center. The design is completed with n_0_ = two observations at the experimental center (runs 15 and 16). Because of the different nature of the experimental design approaches (runs 1-8 and runs 9-14 together with runs 15 and 16), we will separately discuss the results obtained for these two main experimental groups. Biohydrogen production was monitored every 24 h for the duration of all experiments.

**Table 2:** **Central composite experimental design matrix of the three investigated variables, with the total measured H**_**2**_ **production for each of the experimental runs**

**Run number**	**Variable**			**Response**
	**X**_**1**_	***X***_**2**_	**X**_**3**_	**Total hydrogen production mean (mL)**
1	-1	-1	-1	8.66
2	-1	-1	1	9.62
3	-1	1	-1	22.34
4	-1	1	1	25.23
5	1	-1	-1	8.28
6	1	-1	1	8.35
7	1	1	-1	14.67
8	1	1	1	11.92
9	-1.28	0	0	17.67
10	1.28	0	0	10.43
11	0	-1.28	0	2.97
12	0	1.28	0	27.18
13	0	0	-1.28	14.95
14	0	0	1.28	25.92
15	0	0	0	21.08
16	0	0	0	21.66

**Figure 2:**
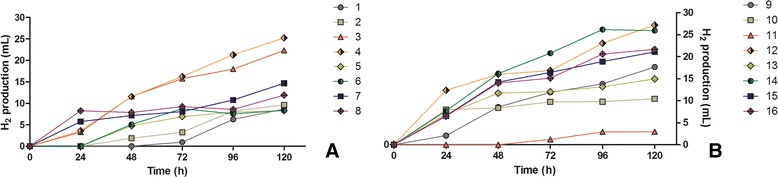
**Biohydrogen evolution measured during the full factorial (A) and additional orthogonal central composite (B) multifactorial experiments.** Clear differences can be observed between the different experimental lines, suggesting a strong influence of the investigated variables on the OF.

Even though the same starting microbial inocula and the same wastewater were used, notable differences in biohydrogen production rates were observed for the factorial portion of the CCD (runs 1-8; Figure 2A). This is the first indication that the selected variables manifest, beyond doubt, a clear influence on the OF in the investigated system. The highest biohydrogen production rates observed during the factorial portion of the CCD were measured during experimental run 4 (a total production of 25.2 mL H_2_ was measured at the end of the experiment), followed closely by experimental run 3 (a total production of 22.3 mL H_2_ was measured at the end of the experiment). The other six experimental runs generated considerably smaller amounts of H_2_, ranging from 14.6 mL (experimental run 7) to 8.2 mL (experimental run 5) H_2_ at the end of the experiment (Figure 2A). Strong similarities in the H_2_ production rates were observed between similar experimental conditions (Table 2). This was particularly true for most of the cases, where only the glucose addition variable (X_3_) differed.

Notable differences in volumetric biohydrogen production were also observed for the supplementary star and central points (experimental runs 9-16; Figure 2B). The highest biohydrogen production rates were obtained for experimental runs 12 and 14 (a total H_2_ production of 27.1 mL and 25.9 mL was measured at the end of the experiments respectively), while the lowest biohydrogen production rate was obtained for experimental run 11 (a total H_2_ production of 2.9 mL was measured at the end of the experiment). Regarding the fermentation conditions of the highest hydrogen-producing experimental run (12), the fermentation temperature (X_1_) as well as the glucose addition (X_3_) values were situated in the center of the experimental model, while the initial pH (*X*_2_) was fixed at a value of 6.74, representing a distance of 1.287 from the experimental center (Table 2). These preliminary findings suggest that the initial value of the environmental pH has a strong influence on the biohydrogen production rate.

The analysis of variance (ANOVA) test performed on the central composite experimental design showed that both linear and quadratic effects of the temperature and pH, as well as their first-order interactions, are statistically significant parameters with regard to the OF (Table 3). However, the main influence of initial glucose addition (X_3_), its quadratic effect, and first-order interactions on biohydrogen production are regarded as statistically insignificant. The lack of fit has a *P*-value slightly higher than α = 0.05, which makes it marginally significant (meaning that it is significant at the 0.10 α - level).

**Table 3:** **ANOVA test performed on the second-order polynomial model used to discriminate between the significant linear (L) and quadratic (Q) effects of the investigated variables, as well as their interactions, for the investigated system and response (biohydrogen production)**

	**Sum of squares (SS)**	**Degree of freedom**	**Mean square**	**F-value**	***P*** **-value**
X_1_-L	270.935*	1*	270.935*	11.63704*	0.001725*
X_1_-Q	260.138*	1*	260.138*	11.17333*	0.002075*
*X*_2_-L	1314.436*	1*	1314.436*	56.45696*	0.000000*
*X*_2_-Q	185.342*	1*	185.342*	7.96069*	0.008033*
X_3_-L	61.992	1	61.992	2.66265	0.112237
X_3_-Q	0.236	1	0.236	0.01013	0.920420
X_1_X_2_	140.261*	1*	140.261*	6.02441*	0.019553*
X_1_X_3_	15.980	1	15.980	0.68634	0.413363
X_2_X_3_	0.291	1	0.291	0.01250	0.911647
Lack of fit	273.054	5	54.611	2.34561	0.062915
Pure error	768.309	33	23.282	**-**	**-**
Total SS	3290.973	47	**-**	**-**	**-**

*Values considered statistically significant.

**Figure 3:**
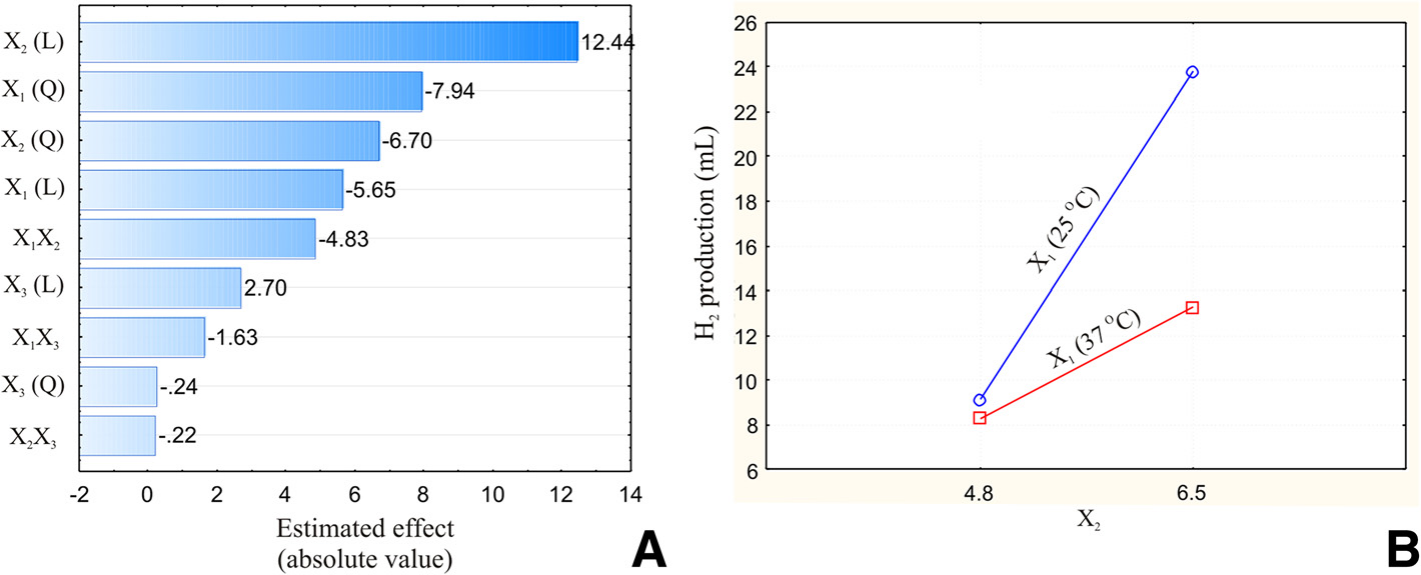
**The effects and the interactions of the investigated variables on the biohydrogen production process.** Pareto histogram depicting the estimated linear (L) and quadratic (Q) effects of each of the analyzed independent variables (in decreasing order of magnitude) on the biohydrogen production rate **(A)**. Analysis of the effect of the interactions between the X_1_ and *X*_2_ variables on biohydrogen production rate **(B)**.

The estimated effects of the IF on the OF together with their order of magnitude were revealed through additional data analysis (Figure 3A). The largest effect on biohydrogen production for the investigated system was caused by a switch in initial pH value from lower to higher values. Fermentation temperature also had a large effect on the OF, followed by the interaction between the fermentation temperature (X_1_) and initial pH value (*X*_2_). The interaction plot for X_1_X_2_ indicates a considerable increase in biohydrogen production at the point when initial pH values move from low to high levels, and temperature is low (Figure 3B). This demonstrates that understanding the interactions among the various process parameters in complex systems is crucial for developing and optimizing an efficient biohydrogen production process.

**Figure 4:**
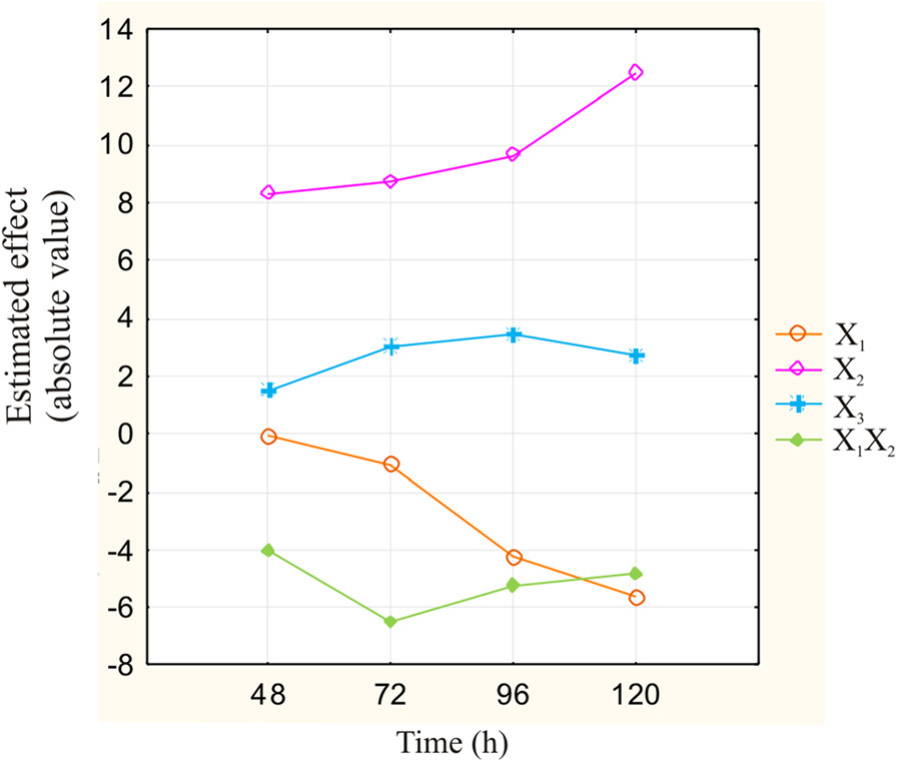
**The evolution of the main effects of the investigated variables and their interactions on the biohydrogen production rate.** The three investigated variables as well as the statistically significant interactions between the operating temperature and the initial pH are represented.

By analyzing the evolution of the main effects throughout the experimental process (120 h), an explicit shift in the direction and intensity of the effect of these variables on the biohydrogen production rate was detected (Figure 4). The main effects of initial pH (*X*_2_) and temperature (X_1_) tended to increase over time, while the effects of other variables, like glucose addition (X_3_), tended to maintain a relatively constant value. These findings emphasize even more the dynamic characteristics of such a complex environment. Thus, the initial environmental conditions as well as the fermentation process settings greatly influenced the development of microbiological processes, and thereby the metabolic end products, in the investigated system. Because of the differences in the direction and intensity of the effects that investigated variables exerted on the objective functions, multiple optimal situations may be identified depending on the point selected in the fermentation development process. Consequently, application of these findings to an industrial-scale continuous biohydrogen fermentation system is crucial for establishing an optimum process development strategy with regard to influent feeding of the system among other factors. These findings particularly support the necessity for a complete exploration of the effects manifested over time on the biohydrogen production process by the different influencing factors.

These crucial insights show that in complex biotechnological systems, both the effect of a particular variable at a specific time on the OF and the global effect of multiple influencing factors together with their different interactions must be considered. In addition, the evolution of the main effects of the IF throughout the biohydrogen fermentation process must be considered. Using these insights, an optimized industrial-scale biohydrogen production system can be successfully designed and operated in a feasible economic context.

### Identification of the optimum conditions for biohydrogen production in the investigated system

**Figure 5:**
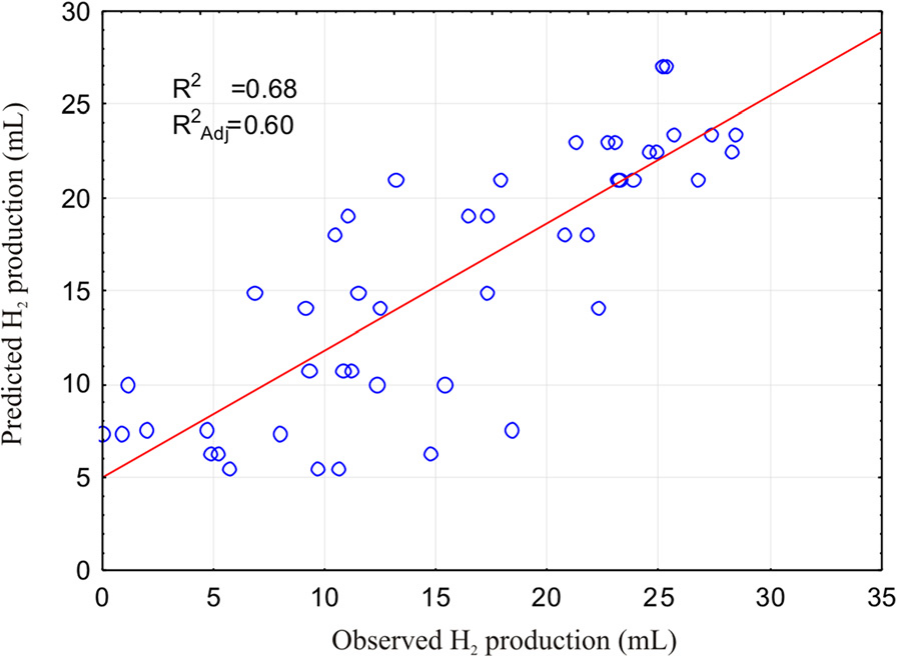
**Modeling the biohydrogen production using beer-brewing wastewater as the fermentation substrate.** Observed versus predicted values of the biohydrogen production process using a complex microbial consortium as the starting inoculum and beer-brewing wastewater as the fermentation substrate.

Once the estimated effects on biohydrogen production rates and their magnitudes were established for each of the analyzed independent variables as well as for their interactions, a strategy to identify the optimum conditions of the investigated system to maximize hydrogen production rates was developed. To understand the specific hydrogen production conditions and to confirm the validity of the statistical experimental strategies applied, a response surface and contour plot methodology was developed. Prediction of H_2_ production at any tested parameter within the range of the applied experimental design is achieved by employing a second-order polynomial regression equation obtained from experimental data (Equation 3 and Figure 5). With respect to the coded factor levels, the second-order model used to fit the experimental data is:

3 $$ Y=20.95-2.83{X}_1+6.22{X}_2+1.35{X}_3-2.42{X}_1{X}_2-0.82{X}_1{X}_3-0.11{X}_2{X}_3-3.97{X}_1^2-3.35{X}_2^2-0.12{X}_3^2 $$

with 60% of the variation in biohydrogen production explained by the model (R^2^_adj_ = 0.60).

**Figure 6:**
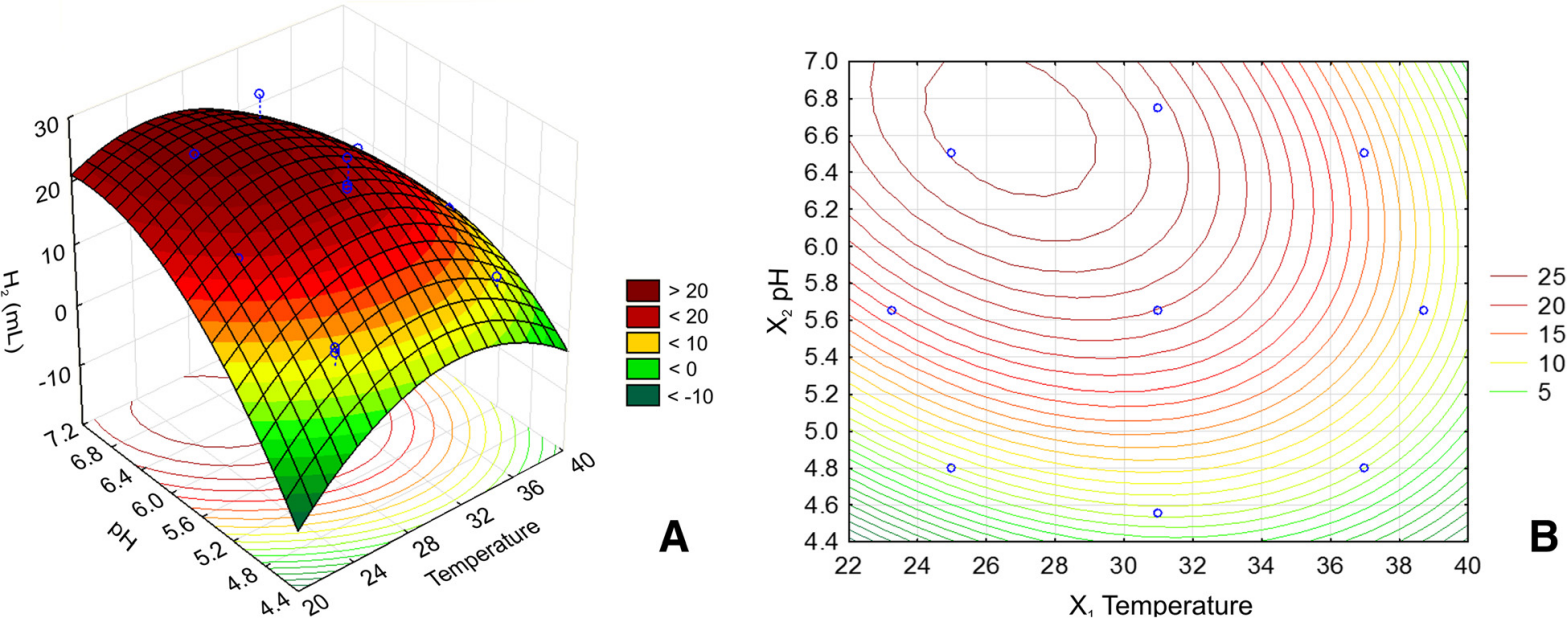
**Prediction of the optimum area for the highest biohydrogen production yields.** Response surface **(A)** and contour plot **(B)** analysis of H_2_ production as a function of temperature and pH, with a constant glucose addition value of 10 g/L.

When considering the observed versus predicted values of biohydrogen production, it can be assumed that the statistical model developed is reasonably accurate. The response surface and contour plot analysis was developed for an initial glucose concentration of 10 g/L (Figure 6). The stationary point was found to be outside the investigated experimental domain. The canonical analysis of the response surface indicates a possible rising ridge, since two of the canonical coefficients are close to zero (λ_1_ = -0.002995; λ_2_ = -0.099786; λ_3_ = -4.65529). In this type of ridge system, inferences about the true surface of the stationary point cannot be drawn because it is outside the region where the model has been fitted. The contour plots for three levels of X_3_ (initial glucose concentration; low, medium, and high) display an optimum around X_1_ = 27°C, *X*_2_ = 6.7, which also indicates that the influence of X_3_ on biohydrogen production in the investigated experimental domain is limited. Therefore, if the initial glucose addition is pooled out from the model (according to Equation 4), considering that it was found statistically insignificant in the investigated domain, the stationary point is found at X_1_ = 26.7°C and *X*_2_ = 6.66, with a predicted optimal value of biohydrogen production of 25.57 mL with a ±95% confidence interval.

4 $$ Y=-343.22+9.05{X}_1+74.42{X}_2-0.47{X}_1{X}_2-0.11{X}_1^2-4.64{X}_2^2 $$

where the coefficients are calculated with uncoded (natural) factor values.

**Figure 7:**
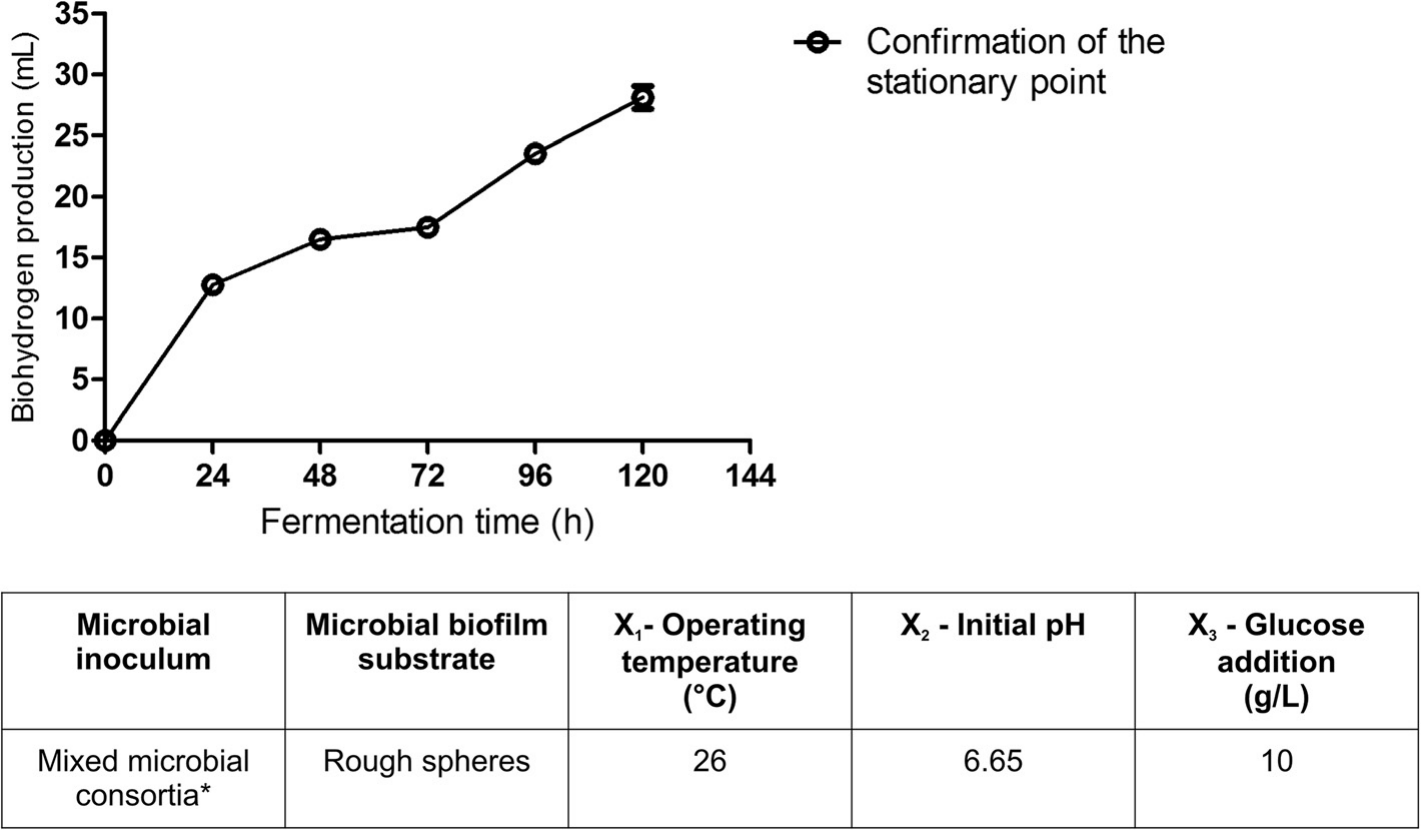
**Confirmation of the predicted stationary point for the investigated system.** The mean of the maximum biohydrogen production yield of 28 mL measured at the end of the confirmation experiments is within the ±95% confidence interval for the predicted optimum area. *The microbial population used as a starting inoculum during the wastewater degradation experiments was subjected to heat pretreatment prior to the inoculation.

The stationary point was verified by carrying out experiments in triplicate. The mean biohydrogen production obtained was 28 mL, and this value was within the ±95% confidence interval for the predicted maximum. Therefore, the new empirical model, with a determination coefficient of R^2^_adj_ = 0.62 and from which all the terms containing the X_3_ independent variable have been pooled out, was able to predict the behavior of the system within the experimental domain (Figure 7).

### Wastewater degradation

During the dark fermentative biohydrogen production process, organic matter is converted from complex long-chain molecules to simple compounds. By using wastewater as a fermentative organic substrate, at least partial biodegradation of this waste can therefore be achieved. Mass spectrometry (MS) was used to evaluate the wastewater biodegradation efficiency. Several organic compounds including lactose, glucose, acetic acid, propionic acid, and furfurol, among others, were identified and monitored during the wastewater degradation experiments. The results allowed direct comparison of the experimental runs by revealing the different degradation rates and metabolic pathways followed by the microbial communities involved.

**Figure 8:**
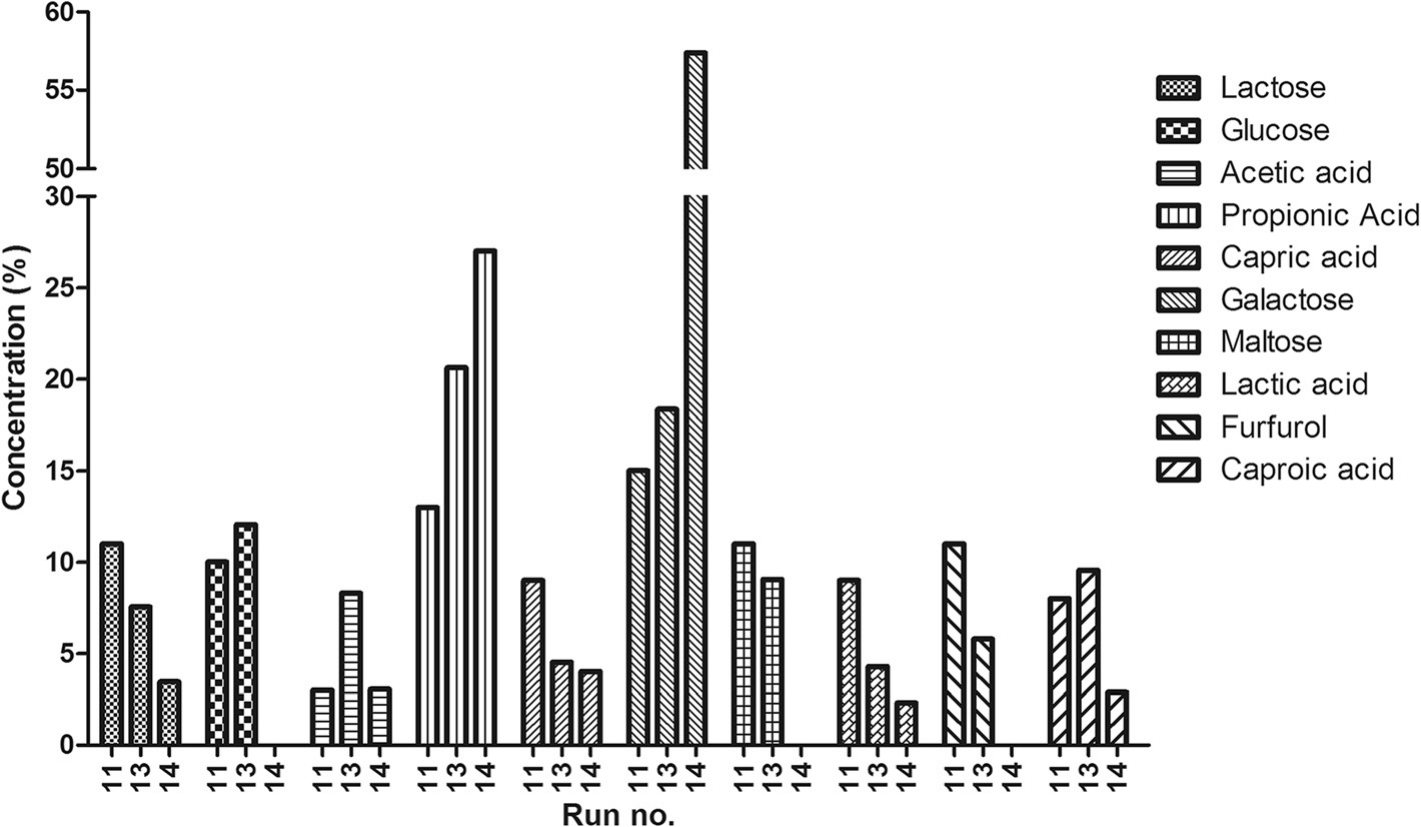
**Wastewater chemical composition at the end of the fermentation period.** Comparison between experimental runs which generated low (experimental run 11), medium (experimental run 13), and high (experimental run 14) hydrogen production rates.

The analysis of three different experimental situations, one with low hydrogen production (experimental run 11), one with medium hydrogen production (experimental run 13), and one with high hydrogen production (experimental run 14), revealed significant differences in the wastewater composition at the end of the biohydrogen fermentation process (Figure 8). As expected, comparison of low and high hydrogen-producing experimental runs revealed a general decrease in concentration of most of the measured components in the fermented wastewater with increasing hydrogen production. This tendency was especially marked for lactose, glucose, capric acid, maltose, lactic acid, furfurol, and caproic acid (Figure 8). These observations suggest that most of the macromolecules in the wastewater are metabolized, with biodegradation efficiency clearly correlating with the amount of hydrogen generated. Some components (glucose, maltose, and furfurol) were fully consumed during fermentation, suggesting that these compounds represent easily accessible energy sources which drive the hydrogen-producing fermentation pathways. At the same time, accumulation of propionic acid and galactose was observed concomitant with the increasing hydrogen yield (Figure 8).

The clear differences between experimental runs observed during the biohydrogen-producing wastewater degradation experiments indicate a strong correlation between the consumption of the organic constituents and hydrogen production rates. This suggests that the technological process developed here has the potential to generate biohydrogen using wastewater as an organic substrate.

### Assessment of microbial community composition

The same mixed microbial consortium was used as a starting inoculum in all experimental runs and stages. This consortium was designed according to our previous research on the ability of various complex consortia to degrade complex organic substrates and simultaneously produce biohydrogen [44]. An organic nutrient-rich habitat (generated during the biological denitrification step at a communal wastewater treatment plant) was sampled in order to isolate the required microbial inoculum. To further increase the biohydrogen-generating abilities of the population, heat pretreatment was applied. As a result, the majority of the spore-forming hydrogen-producing bacteria survived the treatment, while the hydrogen-consuming methanogens were largely eliminated.

To better understand the hydrogen-producing dark fermentation processes, a sequencing-based metagenomic analysis was carried out on samples selected from different experimental runs. This approach allows for a detailed taxonomic and functional characterization of the selected ecosystem as a function of time, which is essential when planning strategies for dark fermentation-based biohydrogen production using wastewater as a fermentative substrate.

To fully understand the experimental model, the complete factorial portion of the CCD was subjected to metagenomic analysis. Additional samples from the center as well as axial experimental points were analyzed metagenomically, with the microbial composition of samples with the lowest and highest H_2_-producing capabilities being investigated. This approach provided valuable insight into the transitions and structural rearrangements occurring in the microbial communities under different fermentation conditions. The influence of these rearrangements on the OF (biohydrogen production rates) was also elucidated.

**Figure 9:**
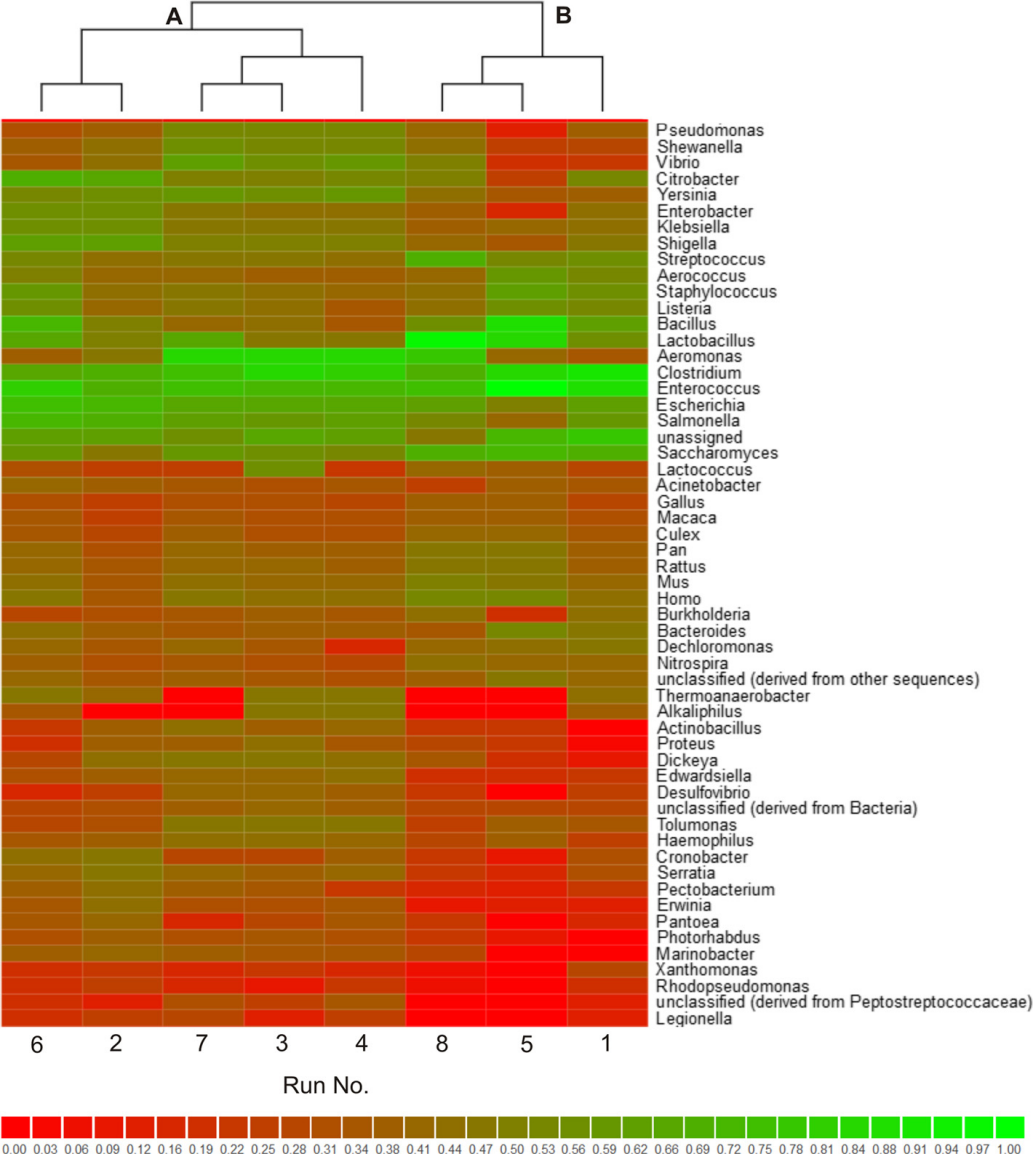
**Partial heatmap calculated for samples from the full factorial experimental design approach (experimental runs 1-8).** The heatmap was redrawn using normalized values clustered by genus using a Bray-Curtis distance metric (divided into clusters **A** and **B** for practical reasons).

The metagenomes extracted from the full factorial experimental design approach (experimental runs 1-8) revealed similarities in the microbial populations collected from the different experimental setups (Figure 9). Two major clusters formed at the genus level. Cluster A contained experimental runs 2, 3, 4, 6, and 7, while cluster B contained experimental runs 1, 5, and 8. In addition, both clusters could be further divided into smaller units. Most of the cluster A microbial communities developed under similar experimental conditions with regard to the initial pH value (*X*_2_) and glucose addition (X_3_). A notable difference between these clustered groups was the fermentation temperature (X_1_). It seems that in the temperature range investigated (25 to 37°C) the microbial composition in each experimental run was similar at the same levels of *X*_2_ and X_3_, regardless of X_1_ levels. Interestingly, the microbial communities located at the closest Bray-Curtis distance (experimental runs 5 and 8), which are isolated from the experimental runs in cluster B, developed under the same temperature conditions (37°C) and different levels of *X*_2_ and X_3_. However, even in this cluster, the microbial community isolated from experimental run 1 developed at the same levels of *X*_2_ and X_3_ but at levels of X_1_ that differed from the community in experimental run 5. These findings suggest a strong correlation between the microenvironmental conditions inside the bioreactor and the different developmental pathways followed by the microbial community.

The cluster formed by samples from experimental runs 3, 4, and 7 displayed the highest H_2_ production potential (22.24 mL, 25.23 mL, and 14.67 mL, respectively). Certain key microorganisms identified in this cluster, such as *Aeromonas* spp*.*, *Clostridium* spp*.*, *Thermoanaerobacter* spp*.,* and *Alkaliphilus* spp., were generally more abundant in experimental runs 3 and 4, while *Bacillus* spp. and *Lactobacillus* spp*.* were poorly represented in these runs (Figure 9).

**Figure 10:**
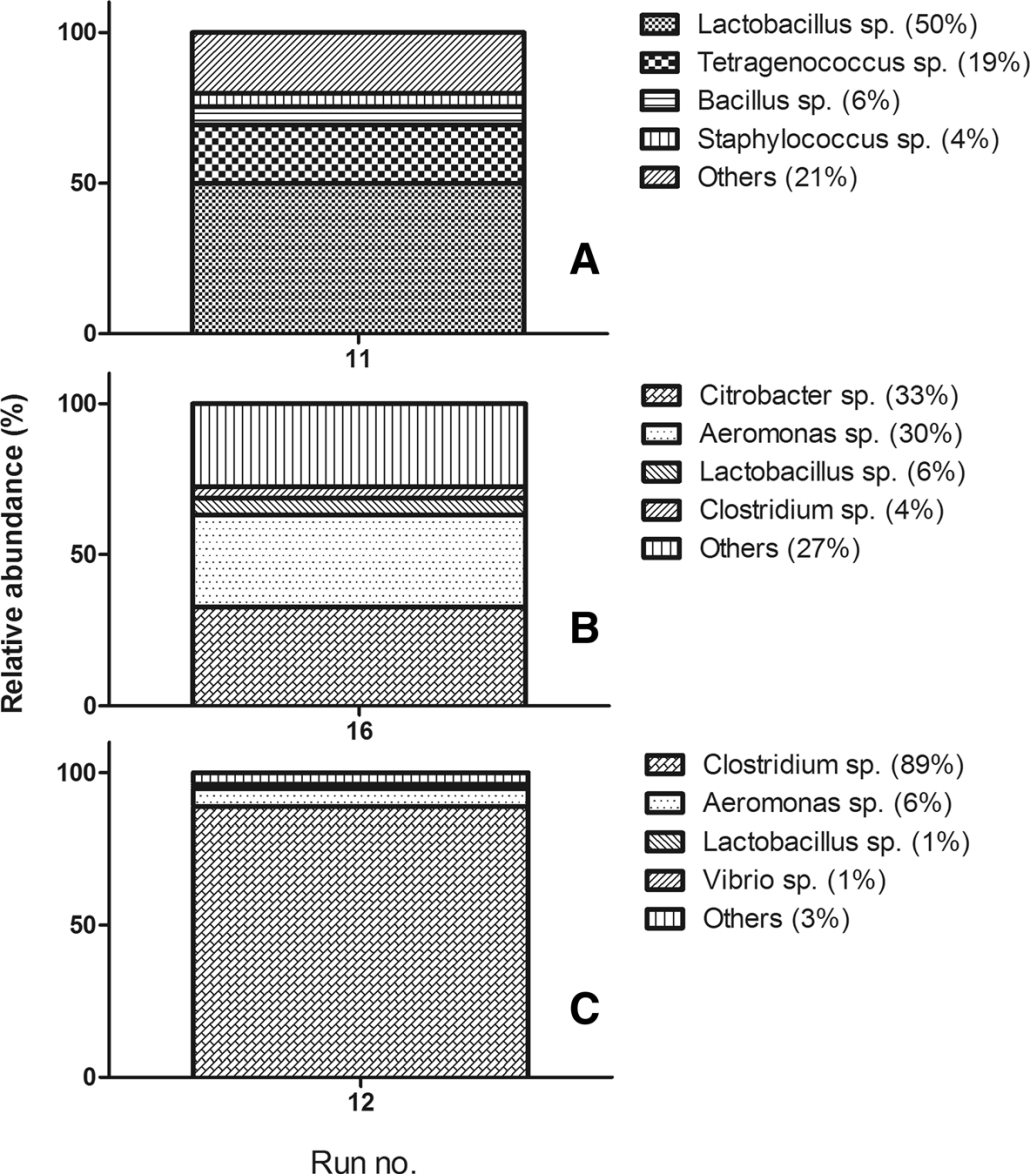
**Microbial population shifts during the biohydrogen production experiments.** Microbial community composition of samples from experimental runs 11 **(A)**, 16 **(B)**, and 12 **(C)**, part of the central composite fractional factorial experimental design approach.

Samples from the supplementary points (star and central points) in the experimental matrix (experimental runs 9-16) were also analyzed using metagenomics, with samples producing the lowest and highest amounts of H_2_ being compared (Figure 2B and Figure 10). Strong correlations were observed between the structural changes within these microbial communities and their biohydrogen-producing potential. The microbial community with the lowest H_2_ production capacity was dominated by *Lactobacillus* spp*.* and *Tetragenococcus* spp*.* (Figure 10A). The microbial consortium which produced moderate quantities of H_2_ was dominated by *Citrobacter* spp*.* and *Aeromonas* spp. (Figure 10B). The microbial population which produced the highest amounts of H_2_ was clearly dominated by *Clostridium* spp. (Figure 10C). A clear shift from a *Lactobacillus* spp*.*-dominated microbial population towards a *Clostridium* spp.-dominated population concomitant with increasing H_2_ production rates was thus observed. Interestingly, the only factor that varied between these three experimental runs was the initial pH value (*X*_2_; Table 2). Therefore, changing only one IF in the investigated system can result in significant changes in the OF (the biohydrogen production rate).

The results obtained from metagenomic investigations have led to a better understanding of the impact of each physicochemical parameter and the interactions between them, all of which are essential for the fermentative biohydrogen production process. To maximize the biohydrogen production potential of a microbial consortium during wastewater degradation, special emphasis has to be made not only on the careful selection and pretreatment of these populations but also on the optimal fermentation conditions for each system.

## Conclusions

A central composite experimental design was used to underline the involvement of the various biological, chemical, and physical factors influencing the fermentative biohydrogen production process. The experiments were performed using a mixed microbial consortium as the starting inoculum and beer-brewing wastewater as the fermentation substrate.

It was demonstrated that the selected variables have a clear influence on the objective function in the investigated system. Both linear and quadratic effects of the fermentation temperature and initial pH, as well as their first-order interactions, were statistically significant with regard to the biohydrogen production rates for the system considered. The largest effect was caused by a change in initial pH value from its lower to its higher level. The fermentation temperature also had a strong effect on the objective function, followed by the interaction between the fermentation temperature (X_1_) and initial pH value (*X*_2_). Analysis of the evolution of these main effects over time during the experiment revealed a strong shift in the direction and intensity of the effect of these variables on the biohydrogen production rate. Because of this, further optimal situations can be identified depending on the point in the fermentation process being investigated. These crucial insights show that this complex biotechnological system is governed by the combined effect of several influencing factors as well as by their interactions with each other.

We successfully used response surface and contour plot methodology to understand the specific hydrogen production conditions of the system under study, and to confirm the validity of the statistical experimental strategies applied. In addition, confirmation experiments yielded a mean biohydrogen production of 28 mL, a value situated within the ±95% confidence interval of the predicted maximum. The new empirical model was thus able to predict the system behavior within the experimental domain.

The mass spectrometry analyses performed on the degraded wastewater during each biohydrogen production experimental run revealed significant differences in the biochemical composition between runs, confirming a strong correlation between the consumption of most of the organic constituents and a high level of hydrogen generation. This indicates the potential of the developed technological process to generate biohydrogen by using wastewater as an organic substrate.

To better understand the microbiological factors driving the dark fermentative biohydrogen production processes in the system studied, high-throughput next generation metagenomic analyses were carried out on samples taken from different experimental runs. These analyses revealed strong correlations between the micro-environmental conditions inside the bioreactors and the developmental pathways taken by the microbial communities, even though the same microbial consortium was used as a starting inoculum in all experimental runs. By analyzing the metagenomes of the lowest and highest H_2_-producing samples, population shifts in hydrogen production potential within the microbial consortia triggered by the different fermentation conditions can be traced. These results have led to a better understanding of the impact which a mixed microbial consortium has on the fermentative biohydrogen production process, and at the same time, the influence of the factors involved on the development of these populations over time.

Together the data generated by the present study reveal a strong interconnection between the investigated variables, as well as a permanent and shifting influence on the biohydrogen production process under the investigated conditions. Only by understanding these phenomena can an optimized industrial-scale biohydrogen production system be successfully designed and operated in a feasible economic context. This will bring us one step closer to a clean, fossil fuel-free future and a developed H_2_-based economy.

## Methods

### Seed inocula

Biological samples were collected from the denitrification step at a municipal sewage wastewater treatment plant. This ecosystem has a high microbial biodiversity consisting of naturally formed populations of microflora suitable for biodegradation of complex organic substrates. Once collected, the samples were stored at 4°C until inoculation.

Prior to inoculating the bioreactors, microbial samples were subjected to a heat pretreatment process at 70°C for one hour as described in our previous work [44]. The purpose of the pre-treatment strategy is to enrich with spore-forming hydrogen-producing microbes as well as to reduce the abundance of hydrogen-consuming microorganisms.

### Bioreactor design and operation

The experimental setup was conducted using wastewater generated by the beer-brewing industry as the fermentation substrate in different experimental combinations as detailed in Table 2. The composition of the wastewater was: total COD - 6558 mg/L, soluble COD - 5066 mg/L, total N - 75.8 mg/L, and total P - 58.4 mg/L. The batch-mode experiments were conducted in 100-mL serum vials with 50 mL of wastewater and 10 mL pretreated sediment samples as a starting inoculum. A granular biofilm support material (sterilized river-bed rocks) was introduced into the bioreactors to increase the contact surface area of the microbial populations. The bottles were capped with rubber septum stoppers and aluminum rings. Incubation was performed at varying temperature conditions for a period of 120 h (Table 2). During the fermentation period, response parameters including biogas production and composition, metabolite concentration, substrate degradation, and total microbial community composition were monitored as described below. All batch experiments were performed in triplicate.

### Statistical experimental design methods

A central composite experimental design approach was used to assess the IF and their effect on the OF (biohydrogen production rate). In the early stage of the experiments, a statistically based full factorial experimental design approach was utilized to determine the influence of the factors under study (fermentation temperature, starting pH value, and glucose addition) on the response (H_2_ production; Table 1). Use of this strategy helped to avoid overlap of different effects and interactions among these variables. The screened factors were chosen based on our previous work and were tested at low, medium, and high levels, coded as -1, 0, and +1 (Table 1) [44]. The factorial portion of the design is a complete 2^3^ factorial with eight runs, which contains all the possible combinations within the defined levels of the investigated variables (runs 1-8; Table 2). In addition, a second statistically based factorial experimental design method was applied in order to obtain a complete understanding of the investigated process. These experimental runs (9- 14) represent additional axial points displayed in a "star pattern" around the center of the design, at α distance of 1.287 from the center, a value that ensures its orthogonality. The design also contains two observations at the experimental center (runs 15 and 16; Table 2). This approach provided N_0_ - 1 degrees of freedom in estimating the experimental error, and at the same time established the estimation precision of the OF (biohydrogen production rate) around the central experimental point. The biohydrogen production rate was monitored every 24 h for the duration of the experiments. Experimental design approaches were developed and analyzed using the Statistica 8 software suite (StatSoft Inc., USA).

### Analytical methods

The quantity and composition of the biogas produced was directly measured by gas chromatography in 24-h intervals using an Agilent Technologies 7890A GC system equipped with a thermal conductivity detector and argon as a carrier gas. The temperatures of the injector, detector, and column were kept at 30°C, 200°C, and 230°C, respectively. An HP PLOTQ column (15 m × 530 mm × 40 mm) was used. Since a concentration gradient of H_2_ gas can form in the headspace, gas samples (0.5 mL) were taken out after mixing of the headspace gas by sparging several times with a gas-tight syringe.

Microbial degradation of wastewater was monitored at the end of the experimental runs by mass spectrometry conducted on a High Capacity Ion Trap Ultra mass spectrometer (HCT Ultra, PTM discovery; Bruker Daltonics, Bremen, Germany). All mass spectra were acquired in the mass range 100 to 3000 m/z, with a scan speed of 2.1 scans/s. Tandem mass spectrometry was carried out by collision-induced dissociation (CID) using He as the collision gas. For MS/MS sequencing, precursor ions were selected within an isolation width of 2 μm. The fully automated process was performed using a NanoMate 400 robot incorporating ESI chip technology (Advion BioSciences, Ithaca, USA) couplet on a High Capacity Ion Trap Ultra mass spectrometer (HCT Ultra, PTM discovery; Bruker Daltonics, Bremen, Germany). The robot was controlled and manipulated by ChipSoft software operated under the Windows system. The position of the electrospray chip was adjusted to the sampling cone potential to give rise to an optimal transfer of the ionic species into the mass spectrometer. To avoid contamination, a glass-coated microtiter plate was used in all experiments. Five microliter aliquots of working sample solutions were loaded onto a 96-well plate. The robot was programmed to aspirate the whole sample volume into the pipette tip followed by 2 μL of air, and then to deliver the sample to the inlet side of the microchip. Each nozzle had an internal diameter of 2.5 μm, and under the given conditions delivered a flow rate of approximately 200 nL/min. The nano-ESI process was initiated by applying voltages of 1.5 to 1.8 kV and a head pressure of 0.5 to 0.7 PSI. After spray initialization, infusion parameters (ESI voltage in the pipette tip, voltage, and desolvation gas flow) were optimized.

Values for ESI capillary, cone potential, and desolvation gas (nitrogen) were optimized to achieve an efficient ionization and to produce the optimum transfer of ions during MS. Measurement parameters were: capillary voltage of 1 kV, contraelectrode voltage (cone voltage) of 60 V, acquisition time 2 min, scan speed 2.1 scans/s, and a mass range of 100 to 3000 m/z. The NanoMate HCT MS system was tuned to operate in the positive ion mode. This technique was chosen because glucide ionization is highly efficient in this mode. The source block maintained at a constant temperature of 80°C provided optimal desolvation of the generated droplets without the need for desolvation gas. To prevent any cross-contamination or carry-over, the pipette tip was ejected and replaced after every sample infusion and MS analysis. All mass spectra were processed using Data Analysis 3.4 software (Bruker Daltonik, Bremen, Germany). The mass spectra were calibrated using sodium iodide. Accurate determination of the average mass was 20 ppm. Samples were dissolved in methanol at a concentration of approximately 5 pmol/μL. At the acquisition time of 2 min, the required volume of sample was approximately 2 pmol, a value indicating a highly sensitive analysis.

### Total DNA extraction from samples

DNA from the complex samples was extracted and purified according to described methods with some modifications [45]. Samples (0.5 g) were extracted with 1.3 mL extraction buffer (100 mM Tris-Cl pH 8.0, 100 mM EDTA pH 8.0, 1.5 M NaCl, 100 mM sodium phosphate pH 8.0, 1% CTAB). After thorough mixing, 7 μL of proteinase K (20.2 mg/mL) was added. After incubation for 45 min, 160 μL 20% SDS was added and mixed by inversion several times with further incubation at 60°C for 1 h with intermittent shaking every 15 min. Samples were centrifuged at 13,000 RPM for 5 min, and the supernatant was transferred into new Eppendorf tubes. The remaining soil pellets were treated three times with 400 μL extraction buffer and 60 μL SDS (20%) and kept at 60°C for 15 min with intermittent shaking every 5 min. Supernatants collected from all four extractions were mixed with an equal quantity of chloroform and isoamyl alcohol (25:24:1). The aqueous layer was separated and precipitated with 0.7 vol isopropanol. After centrifugation at 13,000 RPM for 15 min, the brown pellets were washed with 70% ethanol, dried at room temperature, and dissolved in TE (10 mM Tris-Cl, 1 mM EDTA, pH 8.0).

### Metagenomic characterization of microbial communities

The total DNA from selected samples was prepared for high-throughput next generation sequencing analysis performed on the Ion Torrent PGM platform (Life Technologies). An average of 291.322 sequencing reads were generated for each sample, with a mean read length of 161 nucleotides. Bioinformatic analyses (taxonomic profiling and assessment of metabolic potential) were conducted using the public MG-RAST software package, which is a modified version of RAST (Rapid Annotations based on Subsystem Technology) [46]. The sequence data were compared to M5NR using a maximum e-value of 1 × 10^-5^, a minimum identity of 95%, and a minimum alignment length of 15, measured in amino acids for proteins and base pairs for RNA databases.

## Abbreviations

BioH_2_: biohydrogen
CCD: central composite design
COD: chemical oxygen demand
DOE: design of experiments
IF: influencing factors
OF: objective functions
RAST: Rapid Annotations based on Subsystem Technology
